# A Visualization Template for the Graphical Representation of Sport Injury Antecedents and Consequences Models and Data

**DOI:** 10.3390/jfmk5040087

**Published:** 2020-11-25

**Authors:** Britton W. Brewer, Travis P. Van Brewer

**Affiliations:** 1 Department of Psychology, School of Social Work and Behavioral Sciences, Springfield College, 263 Alden Street, Springfield, MA 01109, USA; 2 Department of Civil and Environmental Engineering, School of Engineering, Tufts University, 200 College Avenue, Medford, MA 02155, USA; tvanbrewer@gmail.com

**Keywords:** antecedents, consequences, athletes, physical injury

## Abstract

A template for visually representing factors affecting and affected by the occurrence of sport injury is presented. The visualization template is designed to facilitate comparison among graphic depictions of models and data pertaining to the antecedents and consequences of sport injury. Innovative aspects and limitations of the visualization template are highlighted, and future applications of the visualization template are discussed.

## 1. Introduction

Injury is ubiquitous in association with sport participation. For example, there are approximately 6 to 8 million sport- and recreation-related injuries documented in Europe [1] and the United States [2] each year. Among the vast array of injuries experienced by athletes are tendinopathies [3,4] and injuries to muscles [5], nerves [6], bones, ligaments, and cartilage [7]. Because of the adverse physical, psychological, social, and economic effects of sport injury [8,9,10], the prediction, prevention, treatment, and rehabilitation of sport injuries and their sequelae have been subjected to extensive scientific inquiry.

To organize knowledge concerning sport injury and facilitate research on the topic, investigators have proposed models that delineate variables and processes associated with sport injury. Some of the models address the antecedents (i.e., etiology) of sport injury [9,11,12,13,14,15,16,17,18,19,20], whereas other models focus on the consequences of sport injury [21,22,23,24,25].

### 1.1. Sport Injury Antecedents Models

Over the past four decades, a variety of models have been proposed (and, in some cases, updated) about the antecedents of sport injury [9,11,12,13,14,15,16,17,18,19,20]. Consistent with the multidisciplinary interest in the phenomenon of sport injury, the models differ in terms of their specific foci, key variables, degree of elaboration, and amount of empirical support. All of the models, however, have the same terminus—the occurrence (or nonoccurrence) of sport injury. For the most part, the models have been represented graphically in two dimensions with key variables (and/or categories of variables) contained in geometric figures (e.g., circles, squares) that are connected by lines and, in some cases, arrows that indicate causal directionality. Despite these commonalities, there has been little consistency in how the models (and the data they have generated) have been represented visually, which makes it difficult to compare and contrast the contents of the models. Furthermore, the reliance on two-dimensional representations (out of necessity for publication purpose, historically) has made it difficult to articulate and foster an appreciation of the complexity of the relationships posited in the models. A more uniform and expansive graphic format would facilitate comparison across sport injury antecedents models and enable articulation of complex aspects of the models.

### 1.2. Sport Injury Consequences Models

As with the antecedents of sport injury, a variety of models have been proposed in which consequences of sport injury are articulated [21,22,23,24,25]. Whereas the sport injury antecedents models have a common endpoint (i.e., sport injury), sport injury consequences models have been applied to an array of disparate outcomes posited to occur after a sport injury has been sustained. Among the focal phenomena targeted in sport injury consequences models are biological healing outcomes [25], psychological responses [21,22], and various rehabilitation outcomes [23,24]. It has long been noted that the variables and categories of variables in sport injury consequences models are highly similar to those in sport injury antecedents models [22], and integration of the two types of models has been advocated [26]. When displayed graphically, sport injury consequences models (and the data they have generated) have generally been represented visually with the same sort of two-dimensional figures with geometric shapes connected by lines that have been used for the sport injury antecedents models. As with the sport injury antecedents models, a more uniform and expansive visual space would facilitate cross-model comparison and highlight the complexity of the models. A graphic format allowing for the visual integration of antecedents and consequences models would have added benefit.

## 2. Sport Injury Antecedents and Consequences Visualization Template Concept

Graphical displays are useful for clear and efficient communication of numerical information. By representing information visually, large and complex data sets can be made more readily understandable. When done well, graphical displays facilitate the comparison of different aspects of data, reveal multiple levels of detail in data, and enable integration of verbal and statistical descriptions of data [27]. Various formats are available to serve as visualization templates onto which projected (i.e., modeled) and obtained data can be plotted. For example, the visual space created by area framed by the *x* and *y* axes in line graphs and scatterplots is ideal for visually representing bivariate relationships, such as the frequency of a particular event over time. For data of greater complexity, more sophisticated visualization templates are needed.

Several features of data pertaining to the antecedents and consequences of sport injury speak to the need for a customized visualization template. First, consistent with the sport injury antecedents and consequences models that guide research on the topic, data sets obtained in research investigations tend to be large and complex. Second, sport injury antecedents and consequences data tend to cut across multiple levels (e.g., biological, psychological). Third, there is no standard graphic format for presenting projected and obtained data concerning sport injury antecedents and consequences models and data.

Given the limitations in existing visual representations of sport injury antecedents and consequences models (e.g., lack of uniformity, spatially constrained, unaccommodating of complexity), the overlap in the components in the two types of models, and the possibility of integrating antecedents and consequences models, the purpose of this concept paper is to develop a visualization template (labelled “sport injury antecedents and consequences visualization template”) for the graphical representation of sport injury antecedents and consequences models and data. It is important to note that the intention is not to propose a new model, but instead to introduce a visualization template that could be used to represent existing and future models and data pertaining to the antecedents and consequences of sport injury. Consistent with advances in data visualization in sport [28,29], health [30,31], and society [32], such a visualization template could help contribute to the development of more comprehensive theoretical models across disciplines; the integration of related but currently separate areas of research; and the emergence of new testable, theoretically-based hypotheses in the sport injury domain. In designing the antecedents and consequences visualization template, several aspirations were kept in mind. Ideally, the visualization template would: (a) have sufficient conceptual space to accommodate extant sport injury antecedents and sport injury consequences models; (b) allow for the addition of new model components and subdivision of existing model components; (c) illustrate direct and indirect (mediated) effects of model components; (d) encourage multidisciplinary inquiry by facilitating theoretical and empirical integration of existing models; and (e) possess the capability of displaying both individual and group (i.e., sample) antecedents and consequences of sport injury.

To accomplish the aims of the sport injury antecedents and consequences visualization template, it is essential to represent two main components: sport injury and clusters of variables effecting and affected by the occurrence of sport injury. Attributes of the sport injury component encompass the type, location, severity, course, duration, chronicity, history, and other key descriptors of sport injury. Drawing from some of the more broadly-focused sport injury antecedents and consequences models [9,10,23], four general categories of antecedents and consequences can be identified: (a) biological, (b) psychological, (c) sociocultural environment, and (d) physical environment. Examples of biological factors include allostatic load, biomechanics, body composition, conditioning level, hydration status, nutritional status, recovery status, and somatic weakness. Examples of psychological factors include anxiety, attentional focus, coping resources, life event stress, mood state, and risky behavior. Features of the sociocultural environment include coach influences, cultural context, media scrutiny, officiating, and social support, whereas features of the physical environment include equipment, playing surface, and weather [9,10,23]. Antecedent and consequence variables are assigned to categories primarily for organizational and theoretical reasons. Because the task of assigning variables to categories is not without ambiguity, however, the boundaries between categories should be considered permeable. For example, although a violent action by an opponent constitutes an injury risk occurring within the physical environment in which the sport is played, the action also constitutes a risk occurring within the sociocultural environment because it is initiated by another person. Presumably, this sort of risk could be “located” near the mutual boundary shared by the sociocultural environment and the physical environment in the antecedents and consequences visualization template.

## 3. Sport Injury Antecedents and Consequences Visualization Template

**Figure 1:**
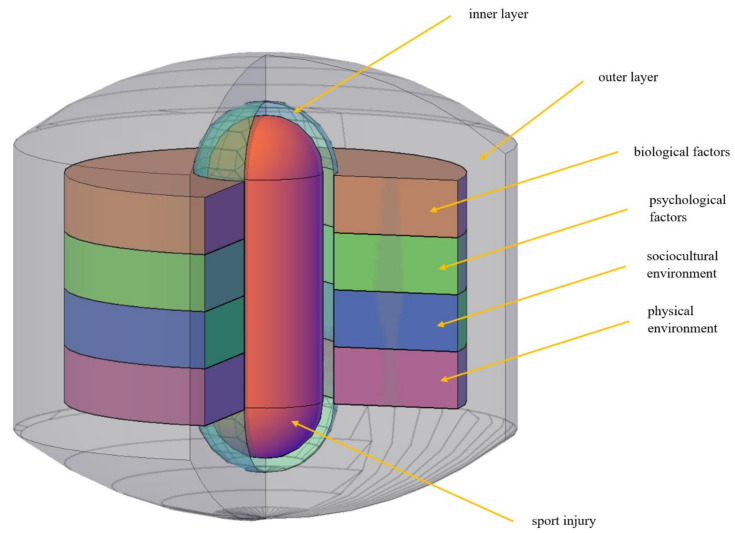
Sport injury antecedents and consequences visualization template.

An external view of the sport injury antecedents and consequences visualization template is presented in Figure 1. A cross-section has been removed to provide a view of the interior of the visualization template. Viewed from the outside in, the first element of the visualization template that one encounters is an outer, epidermis-like layer that contains the remainder of the template. Because there are no limits with respect to the antecedent and consequence variables that can be contained within, the outer layer serves merely as a boundary between what is and what is not part of the visualization template and should not, therefore, be interpreted as an impenetrable border that demarks a closed system.

Proceeding inward, the next element of the visualization template that is encountered is a set of four rings representing the broad categories of antecedent and consequence variables (i.e., biological, psychological, sociocultural environment, physical environment). Variables posited as more distal antecedents or consequences of sport injury can be situated toward the outside edge of a given ring, whereas variables considered to be more proximal antecedents or consequences of sport injury can be located nearer to the inside edges of the rings. The interstitial area between the outer layer and the rings is a space where interactions between variables residing in different rings can be indicated. For antecedent variables, the interactions represented in the outer interstitial area are between stable—as opposed to acute—factors, such as (not easily modifiable) enduring anatomical features, personality characteristics, cultural norms, and customary playing surfaces.

The region on the other side of the interior boundary of the rings is an area that functions somewhat differently for antecedents than for consequences. For the antecedents of sport injury, the area is a zone in which the aggregated risks represented in the four rings “push” and where “acute” interactions between variables from different rings (e.g., momentary inattention when an external source of contact with a vulnerable body part is initiated) can be represented. Chance and other extraneous factors can intervene in this zone to determine whether the accumulated stable and acute risks become, in the terminology of Meeuwisse et al. [18], “inciting events” that lead to injury or mere “events” that do not lead to injury. This area would, therefore, be critical in situations where highly vulnerable athletes escape injury and minimally vulnerable athletes become injured. For the consequences of sport injury, the zone is where interactions between injury characteristics and injury response risk factors can be represented and where injury characteristics “push into”. As with antecedents, chance and other extraneous factors inhabit the zone and, along with the aforementioned injury characteristics and interactions of those characteristics with injury response risk factors, determine whether and/or what consequences occur.

At the center of the visualization template is the focal phenomenon of interest—sport injury. Characteristics of sport injury (e.g., type, course, location, severity, history, duration/chronicity) are represented by a near-infinite number of vertical bands on the face of the capsule-spaced structure. Each band corresponds to a different combination of and is theoretically aligned across the void with a different location on the four rings of antecedents and consequences. It might be tempting to think about the injury structure as akin to a homunculus, with different regions representing different parts of the body. It is possible, however, that an acute, contact-initiated injury to the lower leg, for example, might be located in a nonadjacent and quite distant area of the structure in relation to an overuse injury sustained to the same part of the anatomy. Mapping the injury structure will undoubtedly prove to be one of the formidable challenges in fleshing out the visualization template.

**Figure 2:**
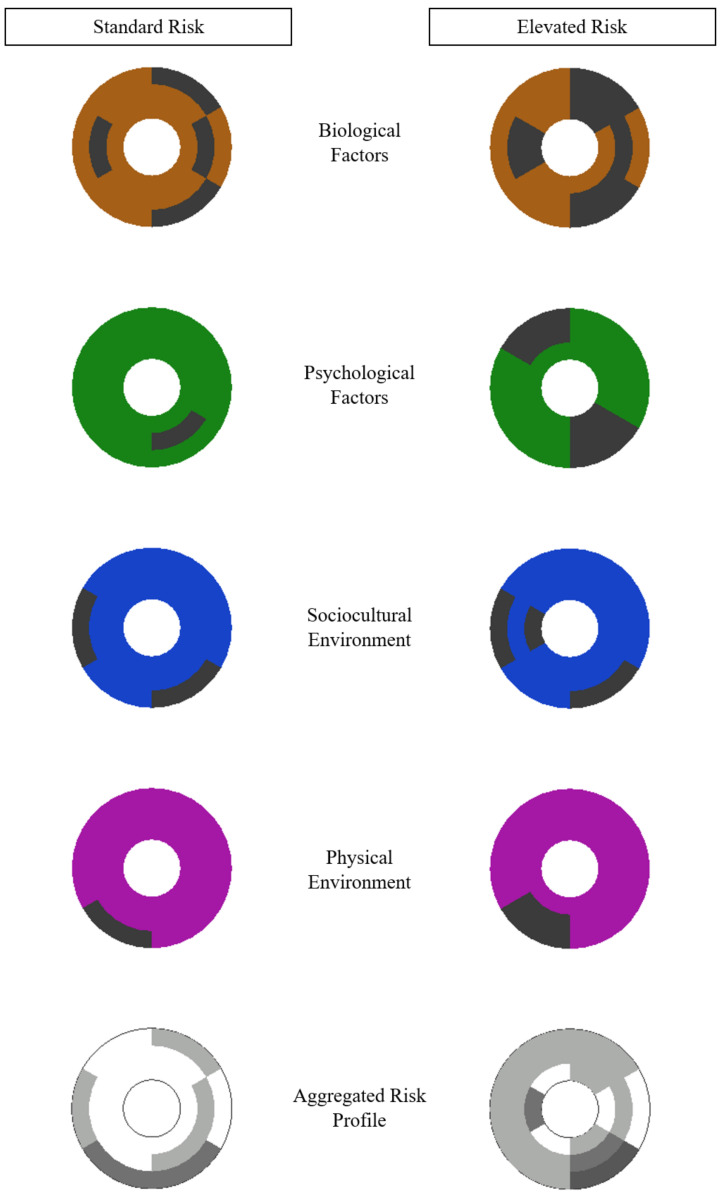
Top view of standard (left column) and elevated (right column) risk in the sport injury antecedents and consequences visualization template. Darkened areas indicate increased risk of injury.

Because the visualization template is a static illustration, multiple depictions are needed to display changes in injury risk and response over time. From an antecedents perspective, Figure 2 shows an hypothetical individual (or a hypothetical population) progressing from a less vulnerable state (on the left side) to a more vulnerable state (on the right side) over time. Cross-sections of the rings of antecedent variables and within-ring shading are shown to offer a sense of the specific areas in which risk of injury increased. The view through all four ring cross-sections stacked on top of each other offers a glimpse of aggregated and concentrated levels of risk bearing on various regions of the injury structure. It should be noted that although Figure 2 is presented and discussed in terms of changes in an individual (or population) over time, it could also correspond to two different individuals (or populations) varying in vulnerability to sport injury.

**Figure 3:**
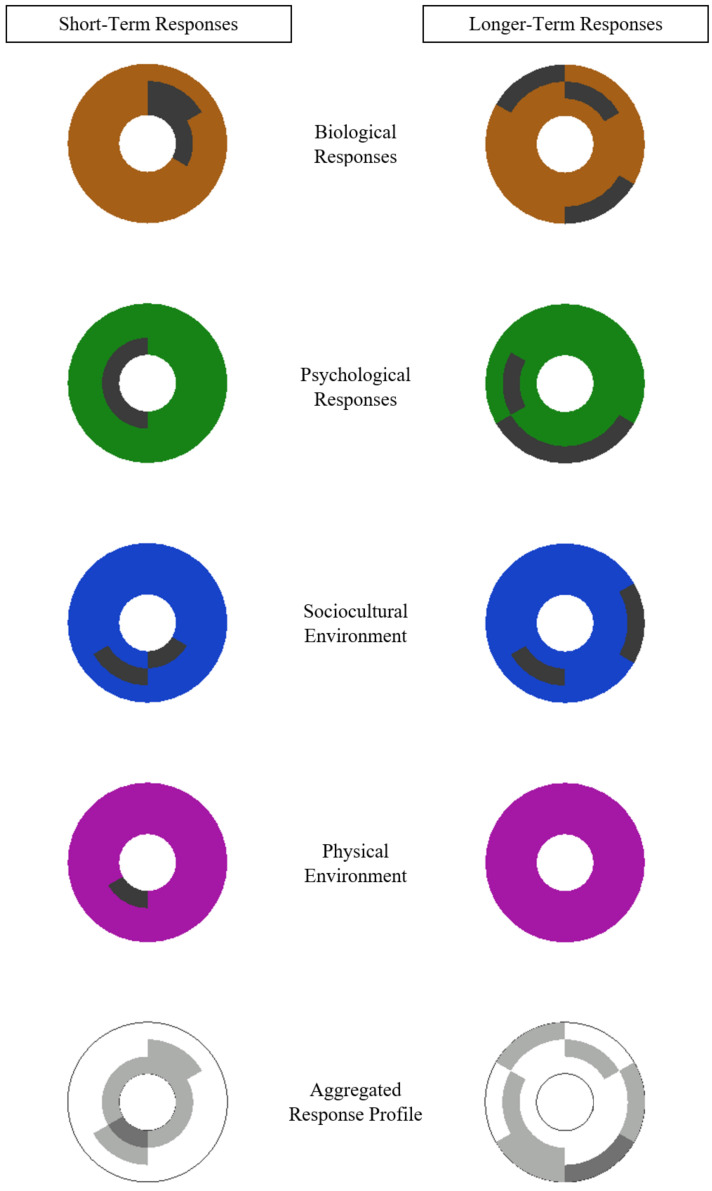
Top view of short-term (left column) and longer-term (right column) consequences in the sport injury antecedents and consequences visualization template. Darkened areas indicate elevated effects of injury.

From a consequences perspective, Figure 3 shows the hypothetical effects of an injury on an individual (or population) at two points in time. The acute effects, which are depicted on the left side of the figure, are more pronounced and more spatially proximal to injury than the longer-term effects illustrated on the right side of the figure. As with Figure 2, Figure 3 could also be used to display the consequences of sport injury for two different individuals (or populations).

## 4. Discussion

In this concept paper, a conceptual visual space is offered for representing models and data pertaining to the antecedents and consequences of sport injury in an inclusive and potentially integrative manner. Although arguments in support of the plausibility and potential utility of the sport injury antecedents and consequences visualization template are given, there are some limitations and challenges concerning the use of the visualization template that should be acknowledged. First, the examples of the visualization template in this concept paper are populated with hypothetical data and largely unspecified variables. A clear next step is to populate the visualization template with actual data and fully specified variables. Second, as noted in the previous section, the visualization template is static and is, therefore, unable to capture (in a single image, anyway) the ways in which the antecedents and consequences of sport injury unfold over time. Representing the temporal aspect of the etiology and sequelae of sport injury is a major challenge that can be addressed partially through multiple time-lapse images of the antecedents and consequences visualization template and more fully through animation. Third, and perhaps above all, the concept of an antecedents and consequences visualization template is novel. It is not customary for scholars to fit (or retrofit) their models into any kind of restrictive space when representing them visually. Consequently, the antecedents and consequences visualization template may be resisted until or unless proof-of-concept is demonstrated.

Despite the limitations and challenges associated with the sport injury antecedents and consequences visualization template, it has the potential for advancing the scientific study of the antecedents and consequences of sport injury in several ways. First, the antecedents and consequences visualization template facilitates the comparison (and possibly promotes integration) of models of the antecedents and consequences of sport injury. Second, the visualization template allows for representation of the full cycle of sport injury, from preinjury on to postinjury. Third, the visualization template encourages interdisciplinary thinking, conceptualization, and, potentially, collaboration and investigation. Fourth, because the visualization template is offered tentatively, as a work in progress, sport injury scholars can modify the framework to suit their needs. Ultimately, it is hoped that the antecedents and consequences visualization template can contribute to a better understanding of sport injury and associated phenomena.
